# Prognostic value of circulating tumour DNA during post‐radiotherapy surveillance in locally advanced esophageal squamous cell carcinoma

**DOI:** 10.1002/ctm2.1116

**Published:** 2022-11-27

**Authors:** Xin Wang, Nuo Yu, Guowei Cheng, Tao Zhang, Jianyang Wang, Lei Deng, Jiao Li, Xiaotian Zhao, Yang Xu, Peng Yang, Na Bai, Yin Li, Nan Bi

**Affiliations:** ^1^ Department of Radiation Oncology, National Cancer Center/National Clinical Research Center for Cancer/Cancer Hospital Chinese Academy of Medical Sciences and Peking Union Medical College Beijing China; ^2^ Department of Radiation Oncology Cancer Hospital of HuanXing Beijing China; ^3^ Geneseeq Research Institute Nanjing Geneseeq Technology Inc. Nanjing China; ^4^ Department of Thoracic Surgery, National Cancer Center/National Clinical Research Center for Cancer/Cancer Hospital Chinese Academy of Medical Sciences and Peking Union Medical College Beijing China

**Keywords:** chemoradiotherapy, circulating tumour DNA, dynamic monitoring, esophageal squamous cell carcinoma, prognosis, radiotherapy

## Abstract

**Background:**

The potential of circulating tumour DNA (ctDNA) as a reliable biomarker for relapse/metastasis early detection and prognosis in esophageal squamous cell carcinoma (ESCC) after radiotherapy/chemoradiotherapy (RT/CRT) initiation requires comprehensive investigation.

**Methods:**

Treatment‐naive locally advanced ESCC patients with available baseline plasma samples were prospectively enrolled from November 2018 to January 2020. RT/CRT was delivered with a simultaneous integrated boost of radiation dose. Serial plasma samples were collected at baseline (T_0_), week 4 of RT/CRT (T_1_), 1‐3 (T_2_) and 3‐6 months post‐RT/CRT (T_3_). ctDNA was analysed using next‐generation sequencing of 474 cancer‐relevant genes.

**Results:**

A total of 128 plasma samples from 40 eligible patients were analysed (median age: 64 [range: 40‐78], 88% males, 95% stage III/IV), and the median follow‐up time was 20.6 months (range: 12.2‐33.3). During the post‐RT/CRT surveillance including 36 patients, radiological progression was observed in 16 patients, and 69% (11/16) had detectable post‐RT/CRT ctDNA prior to radiological progression, with a median lead time of 4.4 months compared with radiological imaging. ctDNA positivity at T_1_ (hazard ratio, HR: 3.60, 95% confidence interval, CI: 1.30‐10.01) or T_2_ (HR: 5.45, 95% CI: 1.72‐17.26) indicated inferior progression‐free survival (PFS). ctDNA clearance between T_0_‐T_1_ (HR: 0.31, 95% CI: 0.08‐1.13) or T_0_‐T_2_ (HR: 0.11; 95% CI: 0.02‐0.61) was associated with relatively favourable PFS. Similar results were obtained when focusing on patients without esophagectomy after RT/CRT. Notably, detectable ctDNA at T_1_ was a potential indicator of high local recurrence risks (HR: 4.43, 95% CI: 1.31‐15.04).

**Conclusions:**

ctDNA was identified as a robust biomarker for early detection of disease progression and post‐RT/CRT prognosis stratification in ESCC. Detectable ctDNA at week 4 of RT/CRT might indicate higher local recurrence risks, implying the potential clinical utility of ctDNA tests in guiding post‐RT/CRT treatments for locoregional control in ESCC.

## Graphical abstract

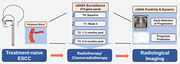

## 1 BACKGROUND

Esophageal cancer (OC), including esophageal squamous cell carcinoma (ESCC) and esophageal adenocarcinoma, ranks tenth in disease incidence (3.1%) and sixth in patient mortality (5.5%) among 36 cancer types worldwide in 2020.^1^ ESCC is the predominant histological type in East Asian countries, such as China and Japan.^2^, ^3^, ^4^ Approximately half of global EC cases are diagnosed in China.^5^ At initial diagnosis, most EC patients are diagnosed with advanced disease, and neoadjuvant radiotherapy/chemoradiotherapy (neo‐RT/CRT) followed by surgery, or definitive RT/CRT serves as the current standard of care.^4^, ^6^ Despite these intensive treatments, the age‐standardised mortality of EC in China ranks top three in 2020 worldwide (http://www.iarc.fr/)^1^, and the 5‐year survival rate in Chinese EC patients is approximately 40%.^7^

Predicting the therapeutic effect of neo‐RT/CRT or definitive RT/CRT in ESCC remains a challenge.^8^ Imaging techniques, including computed tomography (CT), endoscopy, and positron emission tomography‐computed tomography, are widely used in the assessment of tumour burdens in clinical practices. However, imaging methods cannot reliably predict the post‐treatment recurrence in ESCC patients, as the current evaluation criteria in solid tumours criteria are not well suited for hollow organs. A pathologic complete response after neo‐RT/CRT is positively associated with prognosis in EC; however, the pathologic response criteria were exclusively available among patients undergoing resection.^9^, ^10^, ^11^ Thus, there is an unmet need to develop reliable biomarkers for timely detection of recurrent or residual disease, which could facilitate the selection of subsequent therapies and improve the prognosis of ESCC.

Circulating tumour DNA (ctDNA) has been identified as a promising biomarker for tumour diagnosis and disease surveillance. Specifically, ctDNA positivity and dynamics are associated with disease burden in multiple epithelial cancers,^12^, ^13^, ^14^ and has been used to dynamically monitor disease progression in various cancers, including breast cancer,^15^, ^16^ colorectal cancer,^17^ gastric cancer.^18^. In terms of EC, ctDNA is associated with recurrence, metastasis and disease‐specific survival.^6^, ^13^, ^19^, ^20^, ^21^ Nevertheless, these ESCC studies mainly focused on the binary status of ctDNA detection at limited time points, such as pre‐ and/or post‐RT/CRT, and the prognostic value of dynamics in ctDNA has not been well characterised.

Herein, we collected serial peripheral plasma samples from ESCC patients at baseline, during RT/CRT treatment and during post‐RT/CRT disease surveillance. We investigated the association between the longitudinal ctDNA data and tumour relapse/metastasis, and explored the prognostic value of ctDNA monitoring in ESCC.

## 2 METHODS

### 2.1 Study design and participants

In this prospective study, tumour‐naive patients were consecutively recruited at the Cancer Hospital of Chinese Academy of Medical Sciences from November 2018 to January 2020. Inclusion criteria were: (1) aged 18‐80 years; (2) treatment‐naive patients with pathologically confirmed ESCC; (3) clinical stage II‐IVb (stage IVb only with metastatic lymph nodes in the supraclavicular area), according to the 8th edition of the American Joint Committee on Cancer classification; (4) Karnofsky performance score ≥70; (5) available baseline plasma samples collected within 7 days prior to RT/CRT initiation (T_0_). RT doses (planning gross tumour volume [GTV]/planning target volume/fractions) of 44.94 Gy/37.8 Gy/21f and 59.92 Gy/50.4 Gy/28f were delivered for neo‐RT/CRT and definitive RT/CRT, respectively. According to guidelines of Chinese Society of Clinical Oncology,^22^ patients received RT alone, concurrent S‐1 monotherapy, or cisplatin plus paclitaxel‐based doublet chemotherapy, depending on patients’ age, performance status, tolerance of concurrent chemotherapy and/or comorbidities. Patients were evaluated for subsequent esophagectomy at week 4 after initiation of RT/CRT. For patients who were ineligible to receive radical surgery, definitive RT/CRT was performed afterwards.

**FIGURE 1:**
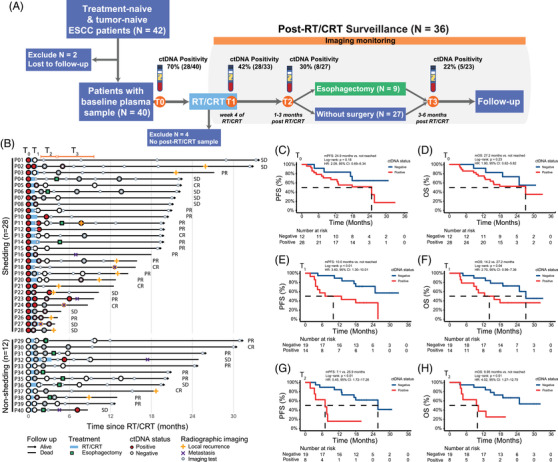
ESCC liquid biopsy surveillance and ctDNA positivity at landmark time points associated with prognosis. (A) In addition to the baseline (T_0_), serial plasma samples were collected at week 4 of RT/CRT (T_1_), 1‐3 months post RT/CRT (T_2_) and 3‐6 months post RT/CRT (T_3_). (B) Excluding 4 patients without post‐treatment ctDNA tests, 16 patients developed recurrence and/or metastasis after RT/CRT, and 11 patients were detected with at least one positive ctDNA results before progression, during post‐RT/CRT disease surveillance. The median lead time was 4.4 (mean: 6.0, 95% CI: 1.3‐10.6) months. (C, D) At T_0_, 28 ctDNA^+^ patients did not show significantly poorer PFS or OS, compared to 12 patients without detectable ctDNA. (E, F) At T_1_, compared to 19 patients with undetectable ctDNA, 14 ctDNA^+^ patients had inferior PFS and OS. (G, H) At T_2_, compared to 19 patients with undetectable ctDNA, 8 ctDNA^+^ patients had inferior PFS and OS

Follow‐up assessments included clinical examination, biochemical tests, barium esophagography, chest/abdominal CT and cervical ultrasound/CT. Patients were followed up once approximately every 3 months for the first 2 years and every 6 months thereafter. Serial plasma samples were collected at three landmark time points, including week 4 of RT/CRT (T_1_), 1‐3 months post‐RT/CRT (T_2_) and 3‐6 months post‐RT/CRT (T_3_) (Figure 1A). At T_1_, around the end of radiotherapy, physicians needed to decide whether the patients should receive further esophagectomy, and ctDNA results at T_1_ could potentially facilitate decision‐making. Patients were suggested booking ctDNA tests simultaneously with the radiological imaging test approximately 1 month after finishing RT/CRT. Owing to the variance of the time of receiving the imaging test, T_2_ was set as 1‐3 months post‐RT/CRT. Considering the feasibility and patient compliance, especially the influence of the Covid‐19 pandemic, T_3_ was determined as 3‐6 months post‐RT/CRT to accommodate more patients. The genetic tests using the Radiotron^®^ panel were conducted in a centralised clinical testing centre (Nanjing Geneseeq Technology Inc., Nanjing, China) according to protocols reviewed and approved by the ethical committee of Cancer Hospital, Chinese Academy of Medical Sciences (NCC2017 G‐079). Details of cell‐free DNA extraction, library construction, next‐generation sequencing and mutation calling are summarised in the Supporting Information.

### 2.2 Variables

The primary outcome was progression‐free survival (PFS), defined as the time from the completion of RT/CRT to relapse and/or metastases. Secondary outcomes included overall survival (OS), locoregional recurrence‐free survival (LRFS) and distant metastasis‐free survival (DMFS). OS was defined as the time from the beginning of RT/CRT to death or the end of follow‐up. LRFS and DMFS were defined as the time from the completion of RT/CRT to local progression and distant metastasis, respectively. The TNM Classification of Malignant Tumors (TNM) was determined following American Joint Committee on Cancer TNM system. For patients with previously detected ctDNA, ctDNA clearance was defined as lacking detectable mutations and copy number variations at one subsequent landmark time point. The ctDNA concentration of one plasma sample was calculated as its cfDNA concentration times maximum variant allele frequency (VAF). The determination of GTV, gross tumour volume of lymph nodes (GTVnd), clinical target volume, and planning GTV was summarised in the Supporting Information.

### 2.3 Statistical analysis

Fisher's exact tests and two‐sample *t*‐tests were performed to compare the frequencies and means between independent subgroups, respectively. Paired two‐sample *t*‐tests were used to compare the means of longitudinal ctDNA concentrations of the same patient. Cohen's kappa coefficient was calculated to measure the agreement of progression detection between ctDNA status and radiological imaging. Kaplan–Meier curves were generated for PFS, OS, LRFS and DMFS, and log‐rank tests were used to compare differences in survival across independent subgroups. Hazard ratios (HR) and 95% confidence intervals (CI) were estimated by Cox proportional hazards models, and the proportionality of hazards was assessed using log(‐log) survival plots. Variables being significant in univariate Cox models or had potential association with prognosis from a clinical point of view were included in multivariable Cox models. Missing values were omitted in the Cox regression model. The median follow‐up time was estimated using the censoring time method.^23^ All quoted *p*‐values were two‐tailed, with <.05 considered to be statistically significant. Data were analysed using R software (version 4.0.3), and the *survival*, *survminer* and *irr* packages.

## 3 RESULTS

### 3.1 Patient overview

A total of 42 treatment‐naive and tumour‐naive ESCC patients were enrolled, and two of them were excluded due to loss to follow‐up. The majority of the remaining 40 patients were males (34/40, 85.0%), and the median age at diagnosis was 64 (range: 40‐78) (Table 1). 57.5% (23/40) and 37.5% (15/40) patients were in stages III and IV, respectively. Thirty‐three patients received CRT with either the platinum‐based chemotherapy regimen (19/40, 47.5%) or S‐1 monotherapy (14/40, 35.0%), while the remaining seven patients (7/40, 17.5%) were treated with RT alone. After completing RT/CRT, 11 patients (11/40, 27.5%) received esophagectomy. The median follow‐up time was 20.6 (range: 12.2‐33.3) months.

**TABLE 1:** Overview of patient demographics and clinical characteristics

Characteristics	Overall (*n* = 40)	Baseline ctDNA^+^ (*n* = 28)	Baseline ctDNA^−^ (*n* = 12)	*p*‐value
Gender, No. (%)				>.99
Female	6 (15.0)	4 (14.3)	2 (16.7)	
Male	34 (85.0)	24 (85.7)	10 (83.3)	
Age at diagnosis, median (range), y	64 (40‐78)	65 (47‐78)	63 (40‐72)	.29
Age at diagnosis, No. (%)				.65
< 70 y	34 (85.0)	23 (82.1)	11 (91.7)	
70+ y	6 (15.0)	5 (17.9)	1 (8.3)	
T stage at initial diagnosis, No. (%)				.65
T1	1 (2.5)	1 (3.6)	0 (0.0)	
T2	2 (5.0)	2 (7.1)	0 (0.0)	
T3	27 (67.5)	17 (60.7)	10 (83.3)	
T4	10 (25.0)	8 (28.6)	2 (16.7)	
N stage at initial diagnosis, No. (%)				.69
N0	2 (5.0)	1 (3.6)	1 (8.3)	
N1	16 (40.0)	12 (42.9)	4 (33.3)	
N2	13 (32.5)	8 (28.6)	5 (41.7)	
N3	9 (22.5)	7 (25.0)	2 (16.7)	
M stage at initial diagnosis, No. (%)				.23
M0	31 (77.5)	20 (71.4)	11 (91.7)	
M1	9 (22.5)	8 (28.6)	1 (8.3)	
Clinical stage at initial diagnosis, No. (%)				.46
II	2 (5.0)	1 (3.6)	1 (8.3)	
III	23 (57.5)	15 (53.6)	8 (66.7)	
IVa	6 (15.0)	4 (14.3)	2 (16.7)	
IVb	9 (22.5)	8 (28.6)	1 (8.3)	
GTV, median (range), cm^3^	37.2 (3.2‐128.2)	40.1 (3.2‐128.2)	28.7 (12.8‐74.2)	.11
GTVnd, median (range), cm^3^	12.72 (0.5‐85.4)	12.72 (0.5‐85.4)	4.2 (0.0‐16.2)	.001^a^
Concurrent chemotherapy, No. (%)				.55
Cisplatin plus paclitaxel	19 (47.5)	12 (42.9)	7 (58.3)	
S‐1 monotherapy	14 (35.0)	11 (39.3)	3 (25.0)	
Radiotherapy alone	7 (17.5)	5 (17.9)	2 (16.7)	
Esophagectomy after radiotherapy, No. (%)				.008^a^
No	29 (72.5)	24 (85.7)	5 (41.7)	
Yes	11 (27.5)	4 (14.3)	7 (58.3)	

Abbreviations: ctDNA^−^, circulating tumour DNA negative; ctDNA^+^, circulating tumour DNA positive; GTV, gross tumour volume; GTVnd, gross tumour volume of lymph nodes.

^a^ Statistically significant.

Baseline ctDNA shedding was detected in 70.0% (28/40) samples, with a median of four (range: 2‐22) mutations per person and a median maximum VAF of 5.95% (range: 0.46%‐46.1%). The most commonly mutated genes in these 28 patients were *TP53* (85.7%), *PRSS3* (21.4%), *CDKN2A* (17.9%), *ART* (14.3%) and *PIK3CA* (14.3%) (Figure S1). Compared to 12 patients without detectable baseline ctDNA, larger GTVnd was observed in patients with baseline ctDNA shedding (mean: 19.6 cm^3^ vs. 5.4 cm^3^, *p =* .001) (Table 1). Post‐RT/CRT esophagectomy was less common among patients with baseline ctDNA shedding (14.3% vs. 58.3%, *p =* .008).

By the end of follow‐up, 19 of 40 (48%) included patients developed radiological disease progression, including 12 with local recurrence only, 4 with distant metastasis only and 3 with both. Median PFS and median OS were 24.9 (95% CI: 17.4‐not reached) and 27.2 (95% CI: 18.0‐not reached) months, respectively.

### 3.2 ctDNA positivity for early detection of disease progression

Excluding four patients without post‐treatment ctDNA tests (P01, P14, P18 and P21), analyses were performed on the remaining 36 patients, of whom 16 (16/36, 44.4%) developed radiological progression by the end of follow‐up (Figure 1B). Eleven of the 36 patients tested positive for ctDNA at least once during post‐RT/CRT surveillance, including nine patients developing positive ctDNA results before radiological diagnosis. The median lead time from detection of ctDNA positivity to radiological progression was 4.4 months (mean: 6.0, 95% CI: 1.3‐10.6). Cohen's kappa coefficient of the agreement between ctDNA positivity and radiological imaging was 0.544 (*p* = .001).

### 3.3 ctDNA positivity at landmark time points indicated poor prognosis

At T_0_, positive ctDNA status was not related to significantly inferior PFS (Figure 1C, HR: 2.09, 95% CI: 0.69‐6.34) or OS (Figure 1D, HR: 1.90, 95% CI: 0.62‐5.82). Also, patients with detectable ctDNA at T_0_ did not have significantly lower objective response rate in comparison with ctDNA^−^ patients (odds ratio: 0.60, 95% CI: 0.11‐2.57, *p* = .51), even though the point estimate was smaller than 1. At T_1_, 33 patients received ctDNA tests, with a lower percentage of ctDNA^+^ patients compared to those tested at T_0_ (42.4% vs. 70.0%, *p =* .03). Patients with detectable ctDNA at T_1_ had worse PFS than ctDNA^−^ patients (Figure 1E, HR: 3.60, 95% CI: 1.30‐10.01). ctDNA positivity at T_1_ was also associated with poorer OS (Figure 1F, HR: 2.70, 95% CI: 0.99‐7.36). Although 13 patients did not receive ctDNA tests at T_2_, the proportion of ctDNA^+^ patients kept declining to 29.6% (8/27). Also, ctDNA positivity at T_2_ indicated inferior PFS (Figure 1G, HR: 5.45, 95% CI: 1.72‐17.26) and OS (Figure 1H, HR: 4.02, 95% CI: 1.27‐12.75). At T_3_, 19 (19/40, 47.5%) patients did not take ctDNA tests due to Covid‐19 pandemic, death, or refusal. The ctDNA^+^ status at T_3_ indicated poorer PFS (Figure S2A, HR: 5.83, 95% CI: 1.53‐22.22) and OS (Figure S2B, HR: 5.74, 95% CI: 1.24‐26.69).

The results of univariate analyses revealed that clinical stage and GTVnd could potentially aid in predicting prognosis (Table 2). Moreover, patients receiving esophagectomy after RT/CRT exhibited a trend of better OS (HR: 0.28, 95% CI: 0.06‐1.20) than those treated with definitive RT/CRT. However, significantly positive association of concurrent chemotherapy with PFS (HR: 0.73, 95% CI: 0.26‐2.08) or OS (HR: 0.90, 95% CI: 0.28‐2.86) was not observed. In multivariable Cox regression models controlling for esophagectomy, clinical stage and GTVnd, ctDNA status at T_1_ remained significantly associated with PFS (HR: 3.35, 95% CI: 1.10‐10.22) and could potentially aid in OS prediction (HR: 2.48, 95% CI: 0.83‐7.37) (Table 3), while ctDNA status at T_2_ might not be a separate prognostic indicator (Table S1). Sensitivity analyses for the prognostic value of ctDNA positivity at T_1_ were performed in patients without surgical treatment, due to the limited number of patients receiving esophagectomy. In multivariable Cox regression models controlling for clinical stage and GTVnd, positive ctDNA status remained negatively associated with PFS (HR: 3.49, 95% CI: 1.13‐10.80) and OS (HR: 2.60, 95% CI: 0.87‐7.83).

**TABLE 2:** Hazard ratios of baseline clinical characteristics estimated by univariate Cox regression models

		PFS		OS	
Characteristics	No. of patients (%)	Hazard ratio (95% CI)	*p*‐value	Hazard ratio (95% CI)	*p*‐value
Gender					
Female	6 (15.0)	Ref	.25	Ref	.16
Male	34 (85.0)	2.42 (0.55‐10.72)		4.30 (0.57‐32.60)	
Age at initial diagnosis					
<70 y	34 (85.0)	Ref	.68	Ref	.83
70+ y	6 (15.0)	0.73 (0.17‐3.21)		0.86 (0.19‐3.75)	
Clinical stage					
II/III	25 (62.5)	Ref	.04^a^	Ref	.02^a^
IV	15 (37.5)	2.61 (1.05‐6.46)		3.01 (1.18‐7.72)	
Concurrent chemotherapy					
No	7 (17.5)	Ref	.55	Ref	.86
Yes	33 (82.5)	0.73 (0.26‐2.08)		0.90 (0.28‐2.86)	
Esophagectomy after radiotherapy					
No	29 (72.5)	Ref	.17	Ref	.09
Yes	11 (27.5)	0.42 (0.12‐1.45)		0.28 (0.06‐1.20)	
GTV					
<37.2 cm^3^	20 (50.0)	Ref	.24	Ref	.08
37.2 + cm^3^	20 (50.0)	1.74 (0.70‐4.36)		2.40 (0.90‐6.41)	
GTVnd					
<9.9 cm^3^	20 (50.0)	Ref	.06	Ref	.06
9.9 + cm^3^	20 (50.0)	2.49 (0.97‐6.37)		2.57 (0.96‐6.85)	

Abbreviations: CI, confidence interval; GTV, gross tumour volume; GTVnd, gross tumour volume of lymph nodes; OS, overall survival; PFS, progression‐free survival; Ref, reference.

^a^ Statistically significant.

**TABLE 3:** Hazard ratios estimated by multivariable Cox regression models at T_1_

		PFS		OS	
Characteristics	No. of patients (%)	Hazard ratio (95% CI)	*p*‐value	Hazard ratio (95% CI)	*p*‐value
ctDNA status at T_1_					
Negative	19 (57.6)	Ref	.03^a^	Ref	.10
Positive	14 (42.4)	3.35 (1.10‐10.22)		2.48 (0.83‐7.37)	
Clinical stage					
II/III	21 (63.6)	Ref	.22	Ref	.29
IV	12 (36.4)	2.18 (0.63‐7.55)		1.92 (0.61‐6.09)	
Esophagectomy after radiotherapy					
No	27 (81.8)	Ref	.68	Ref	.73
Yes	6 (18.2)	0.62 (0.06‐6.12)		0.66 (0.07‐6.70)	
GTVnd					
<9.9 cm^3^	17 (51.5)	Ref	.56	Ref	.29
9.9+ cm^3^	16 (48.5)	1.50 (0.39‐5.79)		2.03 (0.54‐7.63)	

Abbreviations: CI, confidence interval; GTVnd, gross tumour volume of lymph nodes; OS, overall survival; PFS, progression‐free survival; Ref, reference.

^a^ Statistically significant.

### 3.4 Longitudinal ctDNA data for disease monitoring

**FIGURE 2:**
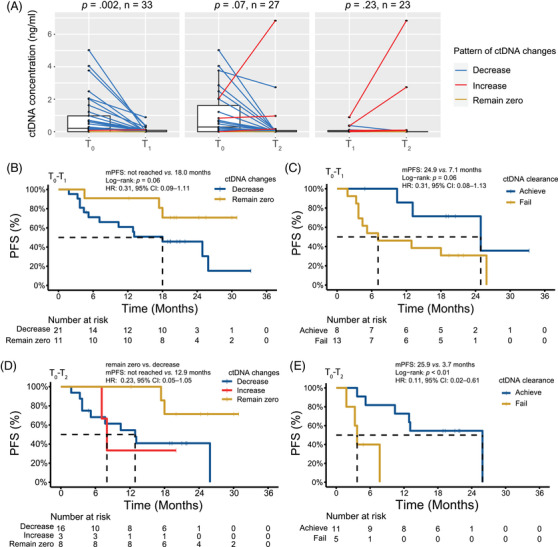
ctDNA dynamics during surveillance and the prognostic value in PFS. (A) At T_1_, 33 patients had significantly lower ctDNA concentration (*p* = .002). At T_2_, a declining trend was observed among 27 patients (*p* = .07), while three patients had increased ctDNA concentration. There was not a clear trend of ctDNA concentration changes between T_1_ and T_2_, with six having decreasing ctDNA concentrations and five having increasing ctDNA concentrations. (B, C) Between T_0_ and T_1_, 11 patients with ctDNA concentration remaining zero had relatively good PFS than 21 patients with decreased ctDNA concentration. Patients achieving ctDNA clearance also showed relatively good PFS, compared to 13 patients who failed to achieve ctDNA clearance. (D, E) Similar results were obtained between T_0_ and T_2_

Between T_0_ and T_1_, the average ctDNA concentration of 33 patients with plasma samples at both time points declined from 0.84 to 0.08 ng/mL (*p* = .002) (Figure 2A). Compared to patients with decreased ctDNA concentrations, patients with ctDNA concentrations remaining zero appeared to have better PFS (Figure 2B, HR: 0.31, 95% CI: 0.09‐1.11) and OS (Figure S3A, HR: 0.32, 95% CI:0.09‐1.13). Moreover, among 21 patients with decreased ctDNA concentrations, a trend of superior PFS (Figure 2C, HR: 0.31, 95% CI: 0.08‐1.13) and OS (Figure S3B, HR: 0.49, 95% CI: 0.15‐1.62) was observed in patients achieving ctDNA clearance, compared to patients failing to achieve ctDNA clearance.

Between T_0_ and T_2_, we obtained similar results for PFS (Figure 2D, HR: 0.23, 95% CI: 0.05‐1.05) and OS (Figure S3C, HR: 0.25, 95% CI: 0.05‐1.21) in patients with ctDNA remaining zero and with decreased ctDNA. We also observed a trend of better PFS (Figure 2E, HR: 0.11, 95% CI: 0.02‐0.61) and OS (Figure S3D, HR: 0.30, 95% CI: 0.08‐1.18) in patients achieving ctDNA clearance, compared to patients without ctDNA clearance.

Between T_1_ and T_2_, five patients with increased ctDNA concentrations showed significantly worse PFS (Figure S3E, HR: 9.09, 95% CI: 1.40‐59.27), compared to six patients with decreased ctDNA concentrations. For these two subgroups, similar results were obtained in OS (Figure S3F, HR: 10.25, 95% CI: 1.56‐67.15). Nevertheless, no significant difference in PFS or OS was detected between patients with decreased and remaining zero ctDNA concentrations.

Sensitivity analyses were performed in patients without surgical treatment. Between T_0_ and T_1_, the differences in PFS (Figure S4A, HR: 0.47, 95% CI: 0.11‐2.11) or OS (Figure S4B, HR: 0.45, 95% CI: 0.10‐2.02) between non‐surgical patients with ctDNA remaining zero and with decreased ctDNA were not significant. Of note, all patients receiving esophagectomy had ctDNA concentration remaining zero from T_0_ to T_1_. Between T_0_ and T_2_, non‐surgical patients with decreased ctDNA appeared to have relatively poor prognosis, when compared to patients with ctDNA remaining zero (PFS, Figure S4C, HR: 0.38, 95% CI: 0.08‐1.79; OS, Figure S4D, HR: 0.35, 95% CI: 0.07‐1.70). Achieving ctDNA clearance was a promising prognostic factor for PFS (Figure S4E, HR: 0.09, 95% CI: 0.01‐0.79) and OS (Figure S4F, HR: 0.11, 95% CI: 0.02‐0.66).

### 3.5 ctDNA positivity indicated the risk of locoregional recurrence

**FIGURE 3:**
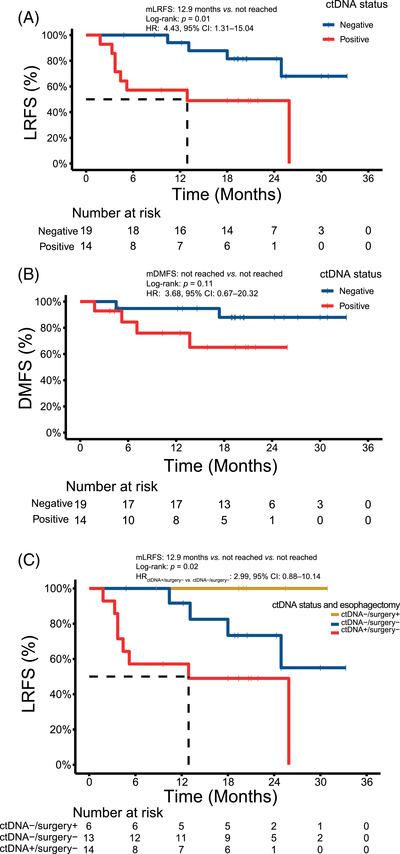
ctDNA status was a potential prognostic indicator of the failure to locoregional control. (A, B) ctDNA positivity at T_1_ was associated with inferior LRFS, while ctDNA positivity at T_1_ was less profoundly associated with DMFS. (C) In the analysis of ctDNA positivity and LRFS stratified according to subsequent esophagectomy, significant differences in LRFS across the three subgroups of patients were observed

At T_1_, ctDNA^+^ was strongly associated with poorer LRFS (Figure 3A, HR: 4.43, 95% CI: 1.31‐15.04), but not DMFS (Figure 3B, HR: 3.68, 95% CI: 0.67‐20.32). No ctDNA^+^ patients subsequently received esophagectomy, while 6 of the 19 ctDNA^−^ patients were treated with esophagectomy. Our data revealed significant differences in LRFS across three subgroups of ctDNA^+^ patients without esophagectomy, ctDNA^−^ patients with esophagectomy, and ctDNA^−^ patients without esophagectomy (Figure S3C, *p =* .02). No local recurrence was observed among six ctDNA^−^ patients receiving esophagectomy. Of the 27 patients receiving definitive RT/CRT, 14 ctDNA^+^ patients had relatively poor LRFS (HR: 2.99, 95% CI: 0.88‐10.14) compared to 13 ctDNA^−^ patients at T_1_.

## 4 DISCUSSION

To the best of our knowledge, this study is the first one comprehensively investigating the prognostic value of longitudinal ctDNA data in ESCC. Detectable post‐RT/CRT ctDNA could indicate radiological progression with a median lead time of 4.4 months. ctDNA positivity at T_1_, T_2_ and T_3_ were negative prognostic factors of PFS and OS, and keeping negative ctDNA status or achieving ctDNA clearance between T_0_ and T_1_/T_2_ potentially indicated a superior prognosis.

In the present study, the detection rate of ctDNA shedding at baseline was 70.0%, close to previous studies (69.8%‐77.1%), and similar frequent mutant genes (such as *TP53*, *CDKN2A* and *PIK3CA*) were identified in our cohort,^13^, ^19^, ^24^ suggesting that our ctDNA detection technique and sequencing data procession were reliable. Intriguingly, our data demonstrated that 18 of 20 (90.0%) patients with large GTVnd were detected with baseline ctDNA shedding, while 10 of 20 (50.0%) patients with small GTVnd were baseline ctDNA^+^, consistent with a previous study suggesting that the level of ctDNA was associated with lymph node involvement.^25^

Compared to the average lead time of 2.8 months in a previous study in the United Sates,^13^ our data suggested that ctDNA positivity during surveillance after RT/CRT preceded radiographic evidence of disease progression by over 4 months. Since a low VAF threshold (0.2%) could increase the sensitivity of ctDNA analysis and bring forward the detection of disease progression, it can be speculated that this discrepancy is partially explained by the lower VAF threshold (0.2%) for retaining somatic mutations in our study. Additionally, our patients were monitored less frequently with imaging analyses during Covid‐19 pandemic, leading to delayed imaging detection of disease progression.^9^ Hence, the potentially overestimated lead time in this study should be interpreted with caution, and further studies with larger sample sizes and shorter interval between each imaging assessment are needed.

A good concordance between ctDNA analyses and radiologic imaging of recurrence was observed in this study, with a relatively high Cohen's kappa coefficient (0.512). Nevertheless, of 36 patients with post‐treatment ctDNA tests, 5 developed imaging confirmed disease progression without prior ctDNA detected, probably resulting from long gaps between their last ctDNA tests and progression. Plasma samples of P03, P19 and P37 were collected 20.5, 5.1 and 19.1 months before progression. Of note, these three patients were not detected with progression in their next imaging tests within approximately 3 months. By contrast, P24 and P28 rapidly developed progression after their last ctDNA tests with intervals of 1.4 and 2.9 months, suggesting that the ultra‐deep sequencing approach were needed, as tumours might not release ctDNA actively after RT/CRT.^10^ Three ctDNA^+^ patients were not identified with radiological progression until the end of follow‐up. P12 harboured mutations with maximum VAF as low as approximately 0.5%, which could explain why this patient was not detected with disease progression; P25 passed away within 3 months after achieving positive ctDNA results, even though no radiological disease progression was identified. Intriguingly, P10 harboured *KMT2C* truncating mutation, a histone modifier gene commonly detected in ESCC patients,^26^ with the VAF of 4.8%, which reminded us that this patient might have potential risk of progression and should be kept under careful observation in the future. Hence, a well‐designed VAF threshold of mutation inclusion is critical to achieve well‐balanced sensitivity and specificity. Distinctive VAF cut‐offs should be set for ESCC driver mutations such as *TP53*, *CCND1*, *NOTCH1* and *MLL2*.^26^, ^27^ The sensitivity of ctDNA detection, depending on sequencing depth, the number of mutations tracked and assay background, could also be improved by designing ESCC specified sequencing panel, and increasing the sequencing depth and panel size. Furthermore, personalised ctDNA assays based on exome or whole genome sequencing of tumour tissues, which have been previously reported for other tumour types, could be a promising approach for ESCC disease surveillance.^28^, ^29^, ^30^

Jia et al. identified that the post‐radiation plasma ctDNA status was an independent prognostic factor for locally advanced ESCC patients, but post‐treatment samples were collected over a prolonged period (56‐310 days post baseline).^5^ In this study, landmark time points were defined clearly, and our data demonstrated that ctDNA^+^ patients at both T_1_ and T_2_ showed poorer PFS and OS than ctDNA^−^ patients. Considering T_1_ as the key time point at which subsequent treatment strategy is determined, and inconsistent conclusions on the benefit from surgery treatment after RT/CRT,^31^, ^32^, ^33^, ^34^ ctDNA status at T_1_ might be used to guide the decision‐making. For ctDNA^−^ patients, organ‐preserved strategy might be an option due to its active influence on quality of life; however, for ctDNA^+^ patients, intensified treatment might be applied to improve the prognosis. Furthermore, we identified a potential association of ctDNA^+^ at T_1_ with inferior LRFS, suggesting ctDNA^+^ patients at T_1_ should take ‘wait and see’ strategy with caution due to high risk of locoregional recurrence. In our study, receiving esophagectomy did not significantly improve LRFS for T_1_ ctDNA^−^ patients, even though none of those receiving esophagectomy developed local relapse by the end of follow‐up. This indicated that surgical treatment after neo‐RT/CRT might not bring considerable benefits to patients with negative ctDNA status at T_1_. However, the relatively small sample size limited our ability to draw a solid conclusion on whether subsequent esophagectomy could be determined based on the ctDNA status at T_1_ alone. The combination of imaging assessment and ctDNA analysis might be a potential option for decision‐making. Therefore, further studies or clinical trials with large sample sizes are warranted to risk‐stratify ESCC patients eligible for optimised and less traumatic management.

Previous ESCC research has not investigated the association of ctDNA status at multiple time points with prognosis. In one study including 97 esophageal adenocarcinoma patients, longitudinal post‐operative ctDNA data were used to predict cancer‐specific survival and disease‐free survival, while the prognostic potential of ctDNA positivity was not separately studied at each landmark time point.^35^ In our study, we observed that the binary status of ctDNA detection at T_2_ and T_3_ served as two potential prognostic factors, suggesting that ESCC patients might have flexible schedules to receive a post‐RT/CRT ctDNA test if a test at T_1_ is not feasible. Furthermore, keeping ctDNA^−^ from baseline or achieving ctDNA clearance between T_0_ and T_1_/T_2_ might indicate better prognosis, consistent with the results reported for other cancers. In one study on postoperative ctDNA surveillance in stages II/III colorectal cancer, complete ctDNA clearance and remaining ctDNA^−^ status were observed exclusively in recurrence‐free patients.^36^ Other studies also suggested that ctDNA clearance of driver and/or passenger mutations during and/or post‐treatment was associated with significantly improved PFS (HR: 0.24, 95% CI:0.13‐0.44; HR: 0.20, 95% CI:0.08‐0.50) and OS (HR: 0.30, 95%, CI:0.14‐0.66; HR: 0.24, 95%, CI:0.08‐0.73) in advanced non‐small cell lung cancer.^37^, ^38^

The limitations of our study should be noted. The primary limitation was the limited sample size and insufficient follow‐up time, especially patients treated with esophagectomy. As a result, a series of trends without statistical significance were observed, and we were unable to collect enough liquid biopsies before disease progression for model fitting, leading to the failure to predict progression risk by personalised dynamic models.^39^ Also, there was a lack of liquid biopsies at disease progression, resulting in the failure to analyse the concordance between ctDNA positivity and radiological progression. Genomic profiles of tissue biopsies and liquid biopsies could neither be compared in this study, as baseline primary tumour tissue samples were not available. Another limitation was the potential confounding effect of combined treatment modality (CRT/RT alone) which could probably influence the association between ctDNA status and prognosis. Moreover, influence of Covid‐19 pandemic resulted in missing data and delayed radiological progression detection, so the prognostic value of ctDNA status or concentration changes were not completely investigated at T_3_. Finally, further exploration of the frequency of imaging assessment combined with ctDNA tests is also required to transform ESCC clinical practice using ctDNA analysis results.

## 5 CONCLUSIONS

ctDNA positivity and dynamics could serve as a robust biomarker for post‐RT/CRT prognosis stratification and early detection of disease progression in ESCC. ctDNA positivity at week 4 of RT/CRT could also potentially indicate a higher risk of locoregional recurrence. Our findings provided a framework for future studies on developing subsequent treatment plans for ESCC patients according to post‐RT/CRT ctDNA status.

## CONFLICT OF INTEREST

Xiaotian Zhao, Yang Xu, Peng Yang and Na Bai are employees of Nanjing Geneseeq Technology Inc., China. The remaining authors have no conflict of interest.

## Supporting information

Table S1. Hazard Ratios Estimated by Multivariable Cox Regression Models at T_2_

Figure S1. Genetic profile of baseline liquid biopsies. Of 40 ESCC patients receiving RT/CRT with or without esophagectomy, 28 had detectable ctDNA at baseline. Genes with at least two alterations (7%) are displayed here. The most frequently altered genes were *TP53* (86%), *PRSS3* (21%), *CDKN2A* (18%), *ATR* (14%), and *PIK3CA* (14%).

Figure S2. Association of ctDNA positivity at T_0_ and T_3_ with prognosis. (A) (B) At T_3_, ctDNA^+^ patients showed significantly poorer PFS or OS, compared to patients without detectable ctDNA, even though only 21 patients had available plasma samples at T_3_.

Figure S3. ctDNA dynamics during surveillance and the prognostic value in OS. (A) (B) Between T_0_ and T_1_, 11 patients with ctDNA concentration remaining zero had relatively good OS than 21 patients with decreased ctDNA concentration. Patients achieving ctDNA clearance also showed relatively good OS, compared to 13 patients who failed to achieve ctDNA clearance. (C) (D) Similar results in OS were obtained between T_0_ and T_2_. (E) (F) Between T_1_ and T_2_, patients with increased ctDNA concentrations showed significantly worse PFS, compared to patients with decreased ctDNA concentrations. similar results were obtained in OS. No significant difference in PFS or OS was detected between patients with decreased and remaining zero ctDNA concentrations.

Figure S4. ctDNA dynamics during surveillance and the prognostic value in non‐surgical patients. (A) (B) Between T_0_ and T_1_, the differences in PFS or OS between non‐surgical patients with ctDNA remaining zero and with decreased ctDNA were not statistically significant. (C) (D) Between T_0_ and T_2_, non‐surgical patients with decreased ctDNA appeared to have relatively poor PFS and OS, when compared to patients with ctDNA remaining zero. (E) (F) Between T_0_ and T_2_, ctDNA clearance was a potential prognostic factor for PFS and OS.
