# Supplementary material for: Prognostic value of circulating tumour DNA during post‐radiotherapy surveillance in locally advanced esophageal squamous cell carcinoma
Source: Clin Transl Med. 2022 Nov 27;12(11):e1116. doi: 10.1002/ctm2.1116 (PMC9702363; doi:10.1002/ctm2.1116)
Supplement: Supplementary file 1 — Table S1. Hazard Ratios Estimated by Multivariable Cox Regression Models at T2 [file CTM2-12-e1116-s002.docx]

*Sample collection, library construction and next-generation sequencing*

Peripheral blood (10 mL) was collected in EDTA-coated tubes, and then centrifuged (at 1800× g for 10 minutes at room temperature) within two hours to separate plasma and leukocyte. Cell-free DNA was extracted from 2 mL plasma by digestion with 100 μL proteinase K buffer for 10 minutes at 37°C followed by purification with the NucleoSpin Plasma XS kit with modified protocols. The purified ctDNA was quantified using a PicoGreen fluorescence assay with the provided lambda DNA standards. Library construction was then performed on 96-well plates using KAPA Hyper DNA Library Prep Kit (KAPA), which contains the reagents for end-repairing, A-tailing, and adapter ligation. Dual-indexed sequencing libraries were amplified by polymerase chain reaction (4–7 cycles), followed by purification. The 5′-biotinylated probe solution was provided as capture probes, targeting 474 cancer-related genes. A sample of (1 μg) of each ctDNA-fragment sequencing library was mixed with 5 μg of human Cot-1 DNA, 5 μg of salmon sperm DNA, and 1 unit adapter-specific blocker DNA in hybridization buffer. The mixture was then heated for 10 minutes at 95°C and held for five minutes at 65°C in the thermocycler. Within the five-minute incubation, the capture probes were added to the mixture, and the solution hybridization was performed for 16–18 hours at 65°C. After hybridization, the captured targets were selected by pulling down the biotinylated probe/target hybrids using streptavidin-coated magnetic beads, and off-target library was removed by rinsing with wash buffers. The PCR master mix was added to amplify the captured library from the washed beads (6–8 cycles). After amplification, the samples were purified using Agencourt AMPure XP beads (Beckman Coulter), quantified using qPCR (KAPA), and sized on bioanalyzer 2100 (Agilent). Libraries were normalized to 2.5 nM and pooled. Enriched libraries were sequenced on Illumina HiSeq 4000 using PE75 V1 Kit (Illumina). Cluster generation and sequencing were performed according to the manufacturer’s protocols. The average coverage depths were at least 3000× for cfDNA, and 100× for normal blood controls.

*Mutation calling and data processing*

Base calling was performed using bcl2fastq v2.16.0.10 (Illumina) to generate sequence reads in FASTQ format (Illumina 1.8+ encoding). Quality control was applied with Trimmomatic [^1^](#_ENREF_1). High quality reads were mapped to the reference human genome (GRCh37-hg19) using modified Burrows-Wheeler Aligner 0.7.12 with BWA-MEM algorithm and default parameters to create SAM files [^2^](#_ENREF_2). Picard 1.119 was used to convert SAM files to compressed BAM files which were then sorted according to chromosome coordinates. The Genome Analysis Toolkit^21^ (GATK, version 3.4-0) was modified and used to locally realign the BAMs files at intervals with indel mismatches and recalibrate base quality scores of reads in BAM files [^3^](#_ENREF_3)^,^[^4^](#_ENREF_4). Single nucleotide variants and short insertions/deletions were identified using VarScan2 with the minimum variant allele frequency (VAF) threshold set at 0.2% and *p*-value threshold for calling variants set at 0.05 to generate Variant Call Format files [^5^](#_ENREF_5). All SNVs/indels were annotated with ANNOVAR, and each SNV/indel was checked manually with the Integrative Genomics Viewer [^6^](#_ENREF_6). Copy number variations were identified using ADTEx 1.0.4 [^7^](#_ENREF_7).

*Determination of radiotherapy-related variables*

Gross tumor volume (GTV) and gross tumor volume of lymph nodes (GTVnd) were determined by barium esophagography, chest/abdominal CT, cervical ultrasound/CT and/or PET-CT. The clinical target volume (CTV) depended on the location of the primary tumor and was irradiated using involved-field radiotherapy. The CTV with a margin of 0.5 cm in three dimensions formed the planning target volume/fractions. The planning gross tumor volume was created by expanding the GTV by 1.0 cm longitudinally and 0.5 cm radially and expanding the GTVnd by a uniform 0.5-cm margin. The typical radiographic contouring for three patients with ESCC in the upper, middle, and lower thirds of the esophagus were published in the protocol of one of our previous phase III trials [^8^](#_ENREF_8).

**Reference**

1. Bolger AM, Lohse M, Usadel B. Trimmomatic: a flexible trimmer for Illumina sequence data. *Bioinformatics*. 2014;30(15):2114-2120.

2. Li H, Durbin R. Fast and accurate short read alignment with Burrows–Wheeler transform. *bioinformatics*. 2009;25(14):1754-1760.

3. McKenna A, Hanna M, Banks E, et al. The Genome Analysis Toolkit: a MapReduce framework for analyzing next-generation DNA sequencing data. *Genome research*. 2010;20(9):1297-1303.

4. Van der Auwera GA, Carneiro MO, Hartl C, et al. From FastQ data to high‐confidence variant calls: the genome analysis toolkit best practices pipeline. *Current protocols in bioinformatics*. 2013;43(1):11.10. 1-11.10. 33.

5. Koboldt DC, Zhang Q, Larson DE, et al. VarScan 2: somatic mutation and copy number alteration discovery in cancer by exome sequencing. *Genome research*. 2012;22(3):568-576.

6. Robinson JT, Thorvaldsdóttir H, Winckler W, et al. Integrative genomics viewer. *Nature biotechnology*. 2011;29(1):24-26.

7. Amarasinghe KC, Li J, Hunter SM, et al. Inferring copy number and genotype in tumour exome data. *BMC genomics*. 2014;15(1):1-12.

8. Li C, Wang X, Wang X, et al. A multicenter phase III study comparing Simultaneous Integrated Boost (SIB) radiotherapy concurrent and consolidated with S-1 versus SIB alone in elderly patients with esophageal and esophagogastric cancer–the 3JECROG P-01 study protocol. *BMC cancer*. 2019;19(1):1-9.
